# Robust Understanding of Robot-Directed Speech Commands Using Sequence to Sequence With Noise Injection

**DOI:** 10.3389/frobt.2019.00144

**Published:** 2020-01-14

**Authors:** Yuuki Tada, Yoshinobu Hagiwara, Hiroki Tanaka, Tadahiro Taniguchi

**Affiliations:** Emergent Systems Laboratory, College of Information Science and Engineering, Ritsumeikan University, Shiga, Japan

**Keywords:** language understanding, service robot, speech recognition, semantic parsing, robot-directed speech detection

## Abstract

This paper describes a new method that enables a service robot to understand spoken commands in a robust manner using off-the-shelf automatic speech recognition (ASR) systems and an encoder-decoder neural network with noise injection. In numerous instances, the understanding of spoken commands in the area of service robotics is modeled as a mapping of speech signals to a sequence of commands that can be understood and performed by a robot. In a conventional approach, speech signals are recognized, and semantic parsing is applied to infer the command sequence from the utterance. However, if errors occur during the process of speech recognition, a conventional semantic parsing method cannot be appropriately applied because most natural language processing methods do not recognize such errors. We propose the use of encoder-decoder neural networks, e.g., sequence to sequence, with noise injection. The noise is injected into phoneme sequences during the training phase of encoder-decoder neural network-based semantic parsing systems. We demonstrate that the use of neural networks with a noise injection can mitigate the negative effects of speech recognition errors in understanding robot-directed speech commands i.e., increase the performance of semantic parsing. We implemented the method and evaluated it using the commands given during a general purpose service robot (GPSR) task, such as a task applied in RoboCup@Home, which is a standard service robot competition for the testing of service robots. The results of the experiment show that the proposed method, namely, sequence to sequence with noise injection (Seq2Seq-NI), outperforms the baseline methods. In addition, Seq2Seq-NI enables a robot to understand a spoken command even when the speech recognition by an off-the-shelf ASR system contains recognition errors. Moreover, in this paper we describe an experiment conducted to evaluate the influence of the injected noise and provide a discussion of the results.

## 1. Introduction

Speech recognition errors are significant in practical tasks provided by service robots. In numerous types of human-robot interactions, it is assumed that the human user will initiate an interaction by giving a spoken command to a service robot at home, in an office, or in a factory. Many studies in the area of robotics and natural language processing (NLP) (Thomason et al., 2015; Misra et al., 2016; Xu et al., 2017) have been conducted to enable a robot to understand the linguistic commands given by human users.

**Figure 1:**
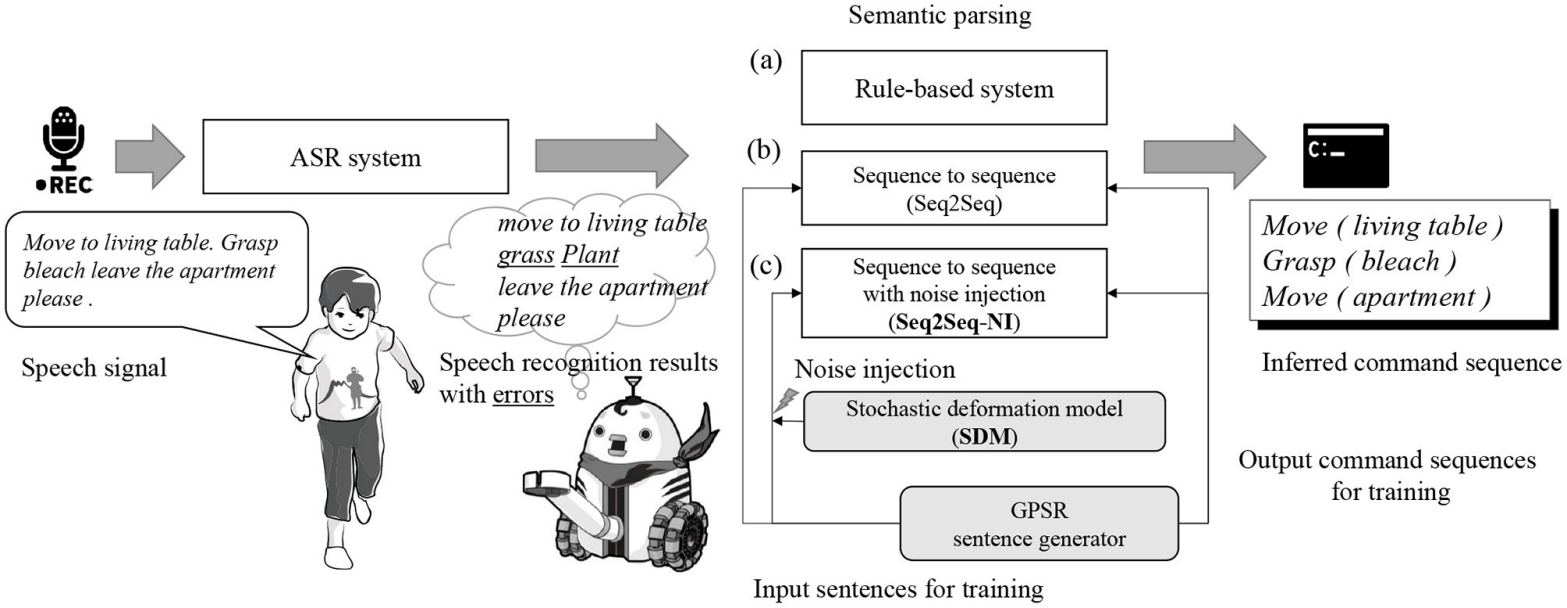
Overview of the process through which a robot understands spoken commands given by a human user. After a speech signal is recognized using ASR and a speech recognition result is obtained, the semantic parser infers the commands intended by the user from the recognized word sequence.

The spoken commands given by a human user are conventionally recognized and understood by a robot in the following manner: First, the robot recognizes a sentence spoken by a human user by applying an automatic speech recognition (ASR) system such as Google Cloud Speech-to-Text API^1^, CMU Sphinx^2^, or Julius^3^. Next, the robot applies syntactic and semantic parsing and determines the sequence of commands that it is expected to carry out. The former part corresponds to the ASR task, and the latter corresponds to the NLP task. The syntactic and semantic parsing for service robots involves a mapping of a recognized sentence to a sequence of commands that is written in an artificial language that can be understood and carried out by the robots (Poon, 2013). An overview of this process is described in Figure 1.

A practical and critical issue in this area is the inevitable occurrence of errors in the results of the speech recognition obtained by the ASR systems, and although significant progress has been made in this field and the performances of such systems have improved considerably, speech recognition errors cannot be completely eliminated. By contrast, conventional studies in the area of NLP have tended to ignore the existence of speech recognition errors. Most methods of semantic parsing in NLP do not have the capability to resolve recognition errors in a sentence, and thus, a robot's understanding of a spoken command may be constrained. The understanding of robot-directed speech commands decreases further with an increase in the number of speech recognition errors. In particular, the environment where a service robot needs to conduct a task may be unfavorable for an ASR system owing to environmental noises involving numerous types of speakers, and because the robot may need to capture the speech signals using a microphone on its body while speakers are at a notable distance from the speaker.

Therefore, the use of off-the-shelf speech recognition systems is a challenge in service robotics when considering that speech recognition errors cannot be eliminated completely even if we use speech recognition systems developed using a state-of-the-art neural network architecture (Amodei et al., 2016; Kim et al., 2017). Improving the performance of language understanding under the conditions through which the robot applies a given speech recognition system is important. The objective of this study is to develop a method of language understanding that enables a robot to comprehend recognized spoken sentences even if the sentences contain several phoneme and word recognition errors.

Off-the-shelf ASR systems usually involve acoustic and language models trained using large speech and text corpora. A language model is a statistical tool that includes information about a vocabulary, and off-the-shelf ASR systems usually contain a large-scale vocabulary to encompass a variety of topics. However, most of these types of systems have little relevance in an actual home, office, or factory environment where the robot needs to perform its tasks. In general, using current technology, the robot can manipulate a limited number of objects, and visit a limited number of places in a domestic environment. For example, in a general purpose service robot (GPSR) task, namely, a task used in RoboCup@Home, which is a standard service robot competition for the testing of service robots, it is assumed that there are a limited numbers of action commands and target objects. This means that the number of command sequences that a service robot needs to map to the speech signal is much smaller than the number of possible sentences generated by the language model, i.e., a dictionary having a large-scale vocabulary. This fact can be used as a semantic constraint when building a robust language understanding system, namely, a semantic parser.

In this study, our primary focus was on GPSR tasks that involve language processing, image processing, and mobile manipulation in an integrative manner. In these tasks, a robot is expected to perform the following commands generated by the GPSR sentence generator^4^. Many studies on service robotics related to GPSR have been reported (Holz et al., 2013; Inamura et al., 2013; Puigbo et al., 2013; Puigbò et al., 2015; Iocchi et al., 2015). In a GPSR task, the sentence is given by a referee as a spoken sentence consisting of three primitive actions, e.g., “Go to the dining table, next, find a stick potato, take it.” During the competition, the site where the robot acts is noisy owing to the size of the audience. Therefore, a clear understanding of the robot-directed speech command is a crucial capability of GPSR.

In the semantic parsing of robot-directed speech, rule-based systems and methods based on symbolic AI have been used for a long time [Figure 1(a)] (Fischer et al., 1996; Lauria et al., 2002; Ljunglöf, 2014; Packard, 2014; Savage et al., 2019). However, the development of rule-based semantic parsing systems requires significant labor, and such systems are usually not robust to noise, i.e., recognition errors are incurred.

Deep neural networks, particularly, recurrent neural networks, have recently been used for semantic parsing in NLP studies. In the same way, an encoder-decoder architecture can be used for semantic parsing, similar to a neural machine translation (Sutskever et al., 2014; Luong et al., 2015). From a mathematical perspective, semantic parsing can be considered a map from a sequence of words to a sequence of semantically understandable symbols including brackets. Some studies on accepting letter or phoneme sequences instead of word sequences as inputs have yielded successful results (Zhang et al., 2015; Gelderloos and Chrupała, 2016; Vosoughi et al., 2016; Xiao and Cho, 2016). Owing to their flexibility, recurrent neural networks are considered to be capable of achieving a morphological analysis inside the network implicitly and applying semantic parsing from sequences of letters or phonemes. Encoder-decoder architecture-based methods for semantic parsing, e.g., sequence to sequence, have produced successful results (Zhou and Xu, 2015; Dušek and Jurcıcek, 2016; Xiao et al., 2016). An encoder-decoder neural network is a continuous, differentiable function compared to rule-based semantic parsing. Sequence to sequence (Seq2Seq) is another candidate method for semantic parsing [Figure 1(b)]. However, these studies did not consider speech recognition errors.

An important concern is mitigating the negative effects of speech recognition errors in language understanding. El Ayadi and Afify (2013) proposed a method for categorizing speech signals, when considering the presence of speech recognition errors, by using word and letter sequence features. Homma et al. (2016) also considered the features of phoneme sequences and described a method for recognizing spoken sentences involving speech recognition errors. These studies consider mapping from a speech recognition result to a category; however, in service robotics, e.g., GPSR, a robot needs to extract more information from spoken commands as the spoken sentence involves various elements of information, including a target object, the goal of a particular movement, the action type, and features of the object (see the right side of Figure 1). For example, when a user says “bring me a dish,” the robot needs to extract elemental actions such as “grasp the dish” and “move to the designated place” by interpreting the given sentence. Methods allowing a robot to interpret a given sentence under the constraint of its set of actions have been studied since the 1970s (Fikes et al., 1972). In this study, we propose a method for converting an input speech recognition result with errors into a sequence of elemental commands by considering a set of actions that can be carried out by the robot.

For this purpose, we prepare a semantic parser that is highly resistant to recognition errors by injecting artificial noise. Noise injection has often been used to increase the robustness of neural networks (Zur et al., 2009; Goodfellow et al., 2016). Bengio et al. (2013) demonstrated the theoretical background of noise injection for an autoencoder. Noda et al. (2014) improved the speech recognition performance by injecting noise into a neural network. Noise injection is regarded as a type of data augmentation that prevents an overfitting and increases the generalization capability of a neural network. Using the *i*-th data sample (*x*_*i*_, *y*_*i*_), where *x*_*i*_ and *y*_*i*_ are the *i*-th input and output, respectively, $\left(x_{i}^{\left[k\right]}=x_{i}+\epsilon_{i}^{\left[k\right]},y_{i}\right)$ can be prepared by injecting noise $\epsilon_{i}^{\left[k\right]}\sim P\left(\epsilon \right)$ into its input. In general, noise injection broadens the receptive field receiving the input to a certain output, i.e., $x_{i}+\epsilon_{i}^{\left[k\right]}$ is mapped to *y*_*i*_. This makes the neural network tolerant to noise and enhances its robustness. The main idea of the approach described in this study is to apply a noise injection scheme to semantic parsing for use in service robotics.

A typical semantic parsing, which has been developed as an NLP method, does not assume speech recognition errors in the input sentences. This means that a robot cannot understand a user's commands unless the speech recognition results are perfectly correct. However, the sentence error rate (SER), which shows the ratio at which a recognized sentence involves at least one error, is much higher than the phoneme error rate (PER) or word error rate (WER). In many cases, the PER and WER are not as high as the SER when using a state-of-the-art off-the-shelf ASR system. Based on this assumption, we applied the noise-injection method to encoder-decoder network-based semantic parsing in which the input is a phoneme sequence. We call this method sequence to sequence with noise injection (Seq2Seq-NI) [Figure 1(c)].

For example, the conventional noise injection approach was often applied to audio or image signals. However, our proposed method injects noise into a phoneme sequence, which is the input data of a semantic parser, in the training datasets. Speech recognition results are variable-length discrete label sequences, and the type of noise to be adopted must be examined. For a suitable noise injection into the phoneme sequences, we used the stochastic deformation model (SDM). The SDM is a stochastic generative model that edits variable-length strings in a probabilistic manner. Taniguchi et al. (2018) developed a mixture of SDMs for the clustering of noisy words.

In this paper, we propose the use of encoder-decoder neural networks, such as sequence to sequence (Sutskever et al., 2014), with noise injection for input into a phoneme sequence during the training phase. We implemented the method and evaluated it using commands applied in GPSR. The results of the experiment showed that the proposed method, i.e., Seq2Seq-NI, outperforms the previous methods. The Seq2Seq-NI enables a robot to understand a spoken command even when the speech recognition results by off-the-shelf ASR systems contain recognition errors. In addition, we conducted an experiment to evaluate the influence of injected noise and discuss the results herein.

The main contributions of this study are as follows: We proposed a Seq2Seq-NI method to infer an appropriate sequence of commands by taking recognized robot-directed speech signals with recognition errors as input and showed that Seq2Seq-NI improves the understanding of a robot-directed command without a mitigation of the speech recognition errors. The remainder of this paper is organized as follows: section 2 describes the Seq2Seq-NI after an introduction to Seq2Seq and SDM. Sections 3 and 4 describe the experiments and demonstrate the effectiveness of Seq2Seq-NI and the effect of the noise injection level. Finally, section 5 provides some concluding remarks.

## 2. Methods

The proposed method, Seq2Seq-NI, is composed of a neural network-based semantic parser using Seq2Seq and a noise generator based on SDM. In this section, we introduce Seq2Seq, Seq2Seq with an attention mechanism and SDM, and finally Seq2Seq-NI.

### 2.1. Seq2Seq for Semantic Parsing

A semantic parser can be developed using Seq2Seq. Semantic parsing of a robot-directed command sentence can be defined as a translation of sentences in languages such as English or Japanese into a sequence of elemental commands for robots. For example, if the parser takes “Please take a bottle and bring it to the living room,” as an input sentence, it should be translated into “Take (bottle) Move (Living room) Place (bottle, Living room table).” For a long time, this type of translation was carried out using a rule-based method; however, it was shown that Seq2Seq, a method for neural machine translation, can be also used for this type of task.

Seq2Seq is a type of neural network with an encoder-decoder architecture that has been mainly used in the field of machine translation (Sutskever et al., 2014). It can map a variable-length sequence of discrete symbols into another variable-length sequence of discrete symbols and determine the relationship between a linguistic sentence, such as a word or letter sequence, and a command sequence for a robot. In a typical case, Seq2Seq consists of two long short-term memory (LSTM) networks, which assume the roles of an encoder and a decoder (Hochreiter and Schmidhuber, 1997). The input information is embedded into an activation pattern of hidden layers by the encoder, and the decoder translates it into a sequence of commands.

**Figure 2:**
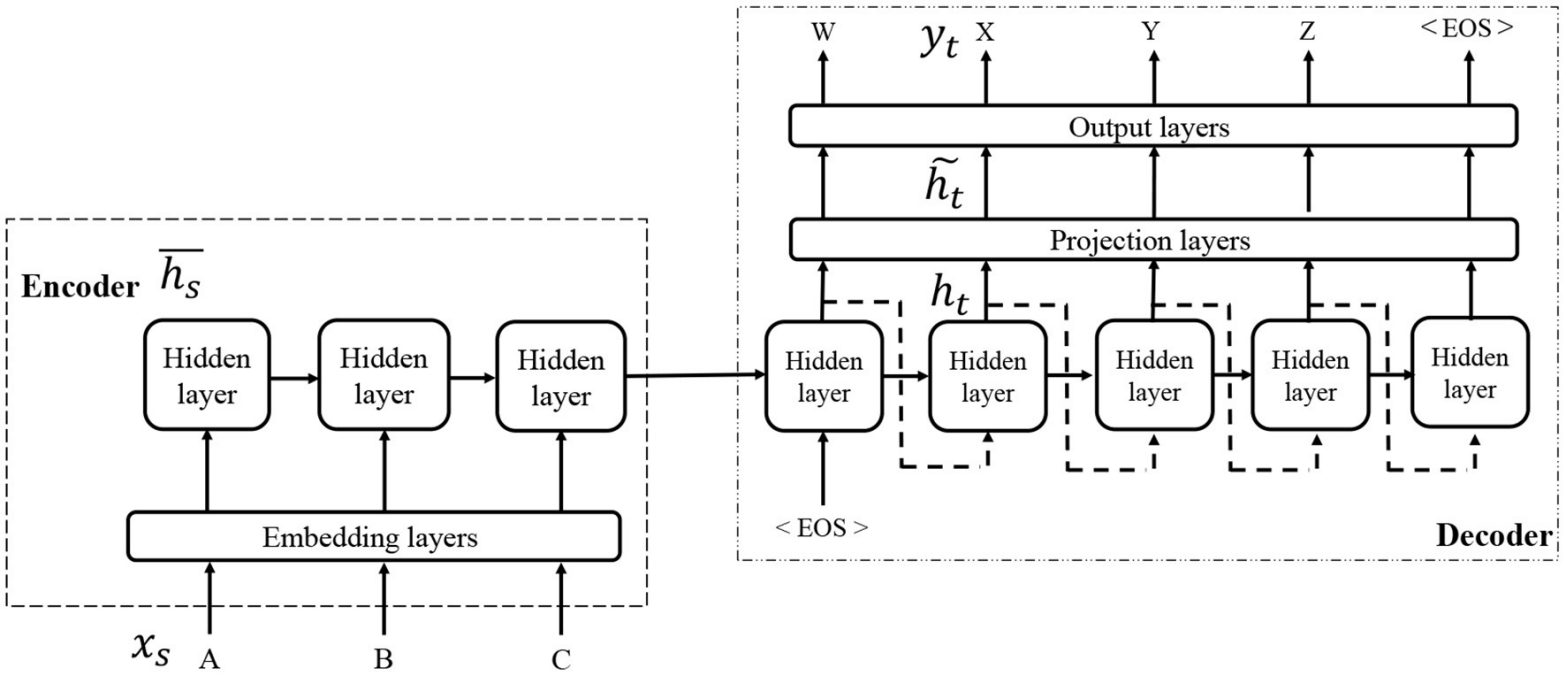
Overview of Seq2Seq.

Figure 2 shows an overview of the network architecture of Seq2Seq. An input sentence *X* = (*x*_1_, …, *x*_*s*_, …) is encoded to a distributed representation $\bar{H}=\left({\bar{h}}_{1},\ldots ,{\bar{h}}_{s},\ldots \right)$ of an encoder. A decoder receives the final vector of the hidden layer of the encoder, and outputs the data *Y* = (*y*_1_, …, *y*_*t*_, …) sequentially. For more details, please refer the original study (Sutskever et al., 2014).

By providing a set of recognized spoken sentence *X* as the input and a correct sequence of commands *Y* as the output, the semantic parser can be trained.

**Figure 3:**
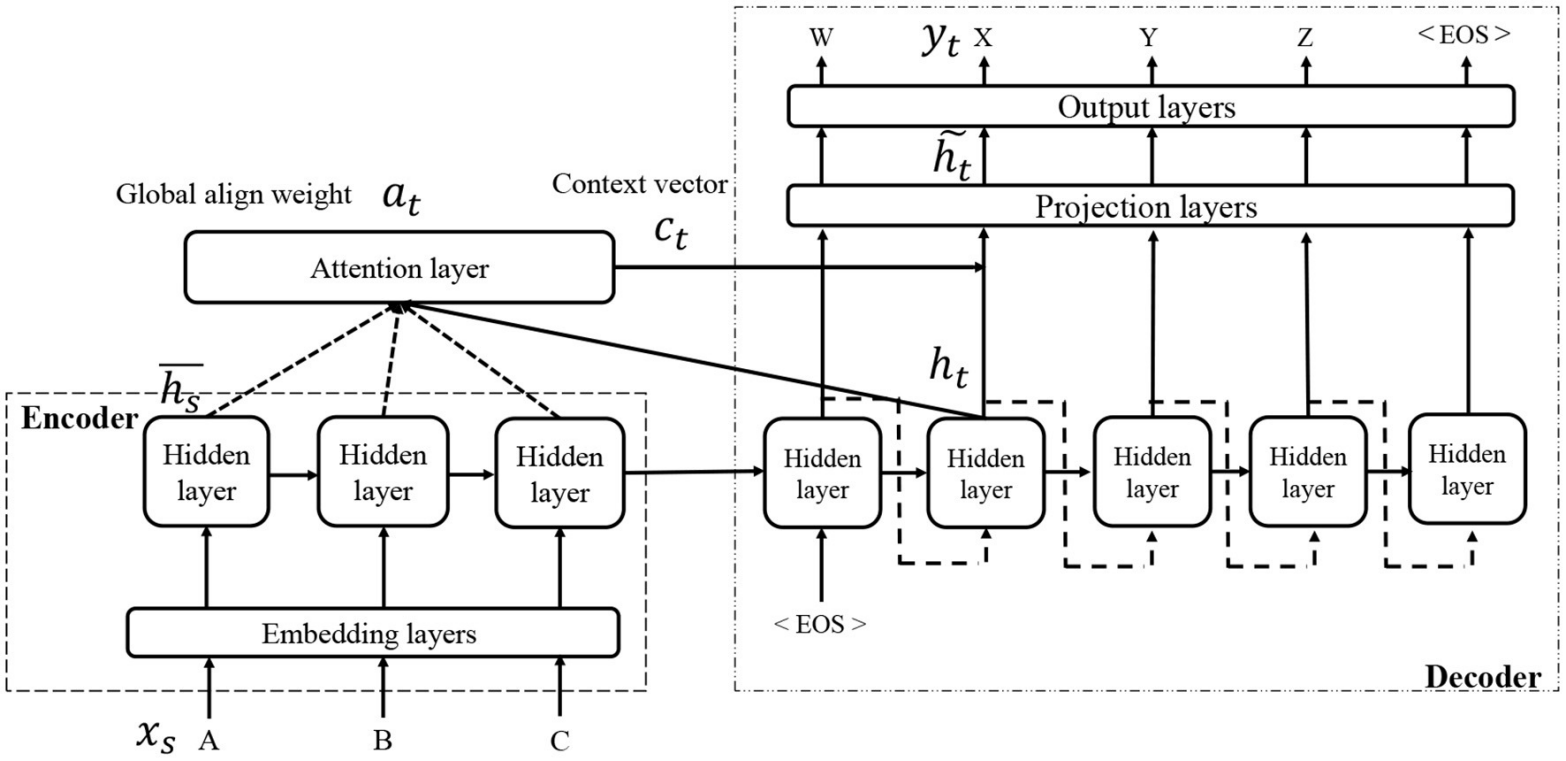
Overview of Seq2Seq with attention.

In the field of neural machine translation, various extensions of Seq2Seq have been proposed, most of which can be used instead of Seq2Seq. One of these is an attention mechanism, and in this study, we consider the use of a local attention model (Luong et al., 2015). Figure 3 presents an overview of the network architecture of Seq2Seq with an attention mechanism. An attention mechanism is the process by which a decoder uses the input information more directly than Seq2Seq, in which all input information must be encoded into a distributed representation. A local attention model is an extension of a global attention model (Bahdanau et al., 2014). For more details, please refer to the original study (Luong et al., 2015).

An open-source implementation of Seq2Seq and the implementation of Seq2Seq using an attention mechanism are available elsewhere^5^, and we used software during the experiments conducted in this study.

### 2.2. SDM for Noise Injection

The SDM involves a stochastic process that deforms a string in a probabilistic manner (Bahl and Jelinek, 1975; Lu and Fu, 1977) and is based on a probabilistic finite state machine (PFSM). It can be regarded as a generative model that provides a mathematical foundation of the edit distance, which is a well-known distance measure of strings, such as a sequence of symbols. We assume that Σ is a set of discrete symbols and Σ^*^ is a set of strings consisting of symbols in Σ. An input sequence, an output sequence, and an edit operation are defined as *X* ∈ Σ^*^, *Y* ∈ Σ^*^, and *T* : Σ^*^ → Σ^*^, respectively.

With SDM, we define three types of elemental operations as follows:

1. Insertion
  $$\begin{array}{l} w_{1}w_{2}\overset{T_{I}}{\mapsto }w_{1}aw_{2},\;a\in \Sigma , \end{array}$$
2. Substitution
  $$\begin{array}{l} w_{1}aw_{2}\overset{T_{s}}{\mapsto }w_{1}bw_{2},\;a,b\in \Sigma , \end{array}$$
3. Deletion
  $$\begin{array}{l} w_{1}aw_{2}\overset{T_{D}}{\mapsto }w_{1}w_{2},\;a\in \Sigma , \end{array}$$

where *w*_1_, *w*_2_ ∈ Σ^*^.

**Figure 4:**
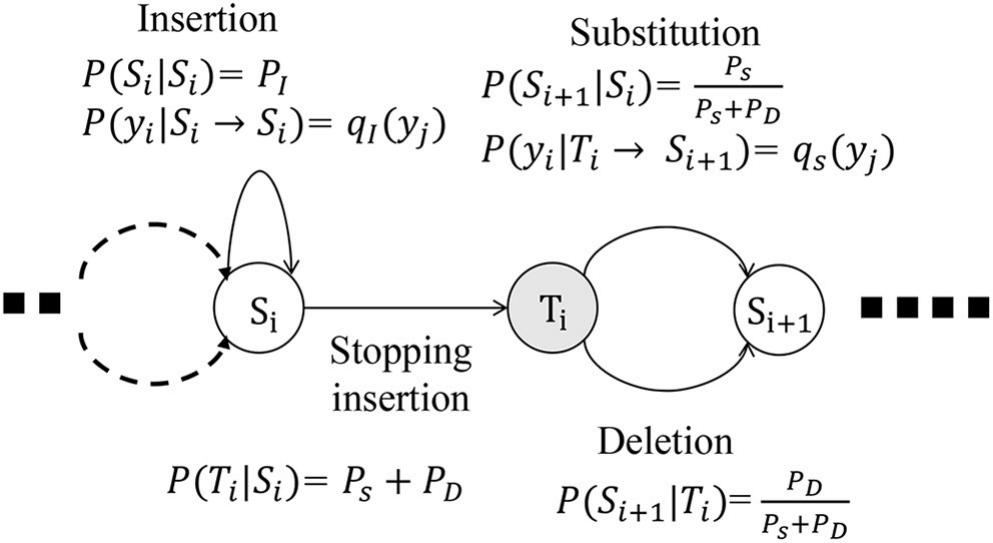
Three types of stochastic operations in PFSM of SDM: insertion, substitution, and deletion.

The three elemental edit operations are illustrated on a PFSM in Figure 4.

Figure 4 describes the state corresponding to the *i*-th input symbol *x*_*i*_. Both the states *S*_*i*_ and *T*_*i*_ correspond to *x*_*i*_. The probability of stochastic deformation satisfies the following equation:

(1) $$\begin{array}{l} P_{I}+P_{D}+P_{S}=1, \end{array}$$

where *P*_*I*_, *P*_*D*_, and *P*_*S*_ are the probabilities for insertion, deletion, and substitution, respectively. Here, *P*(*S*_*i*_|*S*_*i*_) = *P*_*I*_, and the insertion does not change the state of the input. If the probability with which a symbol *y*_*j*_ is inserted is *q*_*I*_(*y*_*j*_; ϕ_*I*_), then *P*(*y*_*j*_|*S*_*i*_ → *S*_*i*_) = *q*_*I*_(*y*_*j*_; ϕ_*I*_), where ϕ_*I*_ is the parameter of the distribution *q*_*I*_. With the probability *P*_*s*_ + *P*_*D*_, the insertion process is terminated and the state transits from *S*_*i*_ to *T*_*i*_.

When the state *T*_*i*_ transits to *S*_*i*+1_, a deletion or substitution is applied.

(2) $$\begin{array}{l} P\left(S_{i+1}|T_{i}\right)=\frac{P_{s}}{P_{s}+P_{D}}. \end{array}$$

The probability that the symbol *x*_*i*_ is substituted by *y*_*j*_ is defined as *P*(*y*_*j*_|*T*_*i*_ → *S*_*i*+1_) = *q*_*s*_(*y*_*j*_|*x*_*i*_; ϕ_*S*_), where ϕ_*S*_ is the parameter of the distribution *q*_*S*_.

The probability that the input symbol *x*_*i*_ is itself the output, i.e., *y*_*j*_ = *x*_*i*_, is regarded as a type of substitution in this model.

Let *P*(*y*_1 : *n*_|*x*_1 : *m*_) denote the probability that the deformation model generates *y*_1 : *n*_ by using *x*_1 : *m*_, where *x*_1 : *m*_ = (*x*_1_, *x*_2_, …, *x*_*m*_), and *y*_1 : *n*_ = (*y*_1_, *y*_2_, …, *y*_*n*_). The probability can be calculated by applying a recursive operation as follows:

(3) $$\begin{array}{l} P\left(y_{1:1}|x_{1:1}\right)=\;1, \end{array}$$

(4) $$\begin{array}{l} P\left(y_{1:1}|x_{1:i+1}\right)=\;P\left(y_{1:1}|x_{1:i}\right)\cdot P_{D}\;\left(i=1,\ldots ,m\right), \end{array}$$

(5) $$\begin{array}{l} P\left(y_{1:j+1}|x_{1:1}\right)=\;P\left(y_{1:j}|x_{1:1}\right)\cdot P_{I}\cdot q_{I}\left(y_{j+1};\phi_{I}\right)\;\left(j=1,\ldots ,n\right), \end{array}$$

(6) $$\begin{array}{l} P\left(y_{1:j+1}|x_{1:i+1}\right)=\;P\left(y_{1:j}|x_{1:i+1}\right)\cdot P_{I}\cdot q_{I}\left(y_{j+1};\phi_{I}\right)\\ \;+P\left(y_{1:j+1}|x_{1:i}\right)\cdot P_{D} \end{array}$$

(7) $$\begin{array}{l} +P\left(y_{1:j}|x_{1:i}\right)\cdot P_{S}\cdot q_{S}\left(y_{j+1}|x_{i+1};\phi_{S}\right). \end{array}$$

By using the SDM, we can inject noise into an input string in a synthetic manner. We can define the stochastic process in the SDM as follows:

(8) $$\begin{array}{l} y\sim SDM\left(x;\theta \right), \end{array}$$

where θ = {*P*_*I*_, *P*_*D*_, *P*_*S*_, ϕ_*I*_, ϕ_*S*_} is a set of parameters of the SDM.

### 2.3. Seq2Seq-NI

Seq2Seq-NI is a semantic parsing method using Seq2Seq and noise-injected input data for training. We assume that huge amounts of real speech signals and their recognition results cannot be obtained; however, a large number of possible sentences and their semantic parsing results can be generated. We assume that a sentence, i.e., a word sequence, output from an off-the-shelf ASR system with recognition errors, is still phonologically similar to the original sentence. Therefore, our method injects phoneme-level noise into the recognized phoneme sequences for data augmentation^6^. In the GPSR task, a sentence generator generates examples of sentences and their meaning, i.e., the correct parsing results. We assume *X* = {*X*_1_, …, *X*_*D*_} to be the generated sentences and $Y^{*}=\left\{Y_{1}^{*},\ldots ,Y_{D}^{*}\right\}$ to be the correct parsing results. We can obtain the augmented training data by injecting noise using the SDM, i.e., $X_{d}^{\left[k\right]}\sim SDM\left(X_{d};\theta \right)$. A synthetic dataset that considers the speech recognition errors, $D={\left\{X_{d}^{\left[k\right]},Y_{d}\right\}}_{k,d}$, is obtained. In Seq2Seq-NI, the Seq2Seq model is trained using the noise injected data *D*. Such noise injected training data are expected to increase the robustness of the semantic parser.

## 3. Experiment 1: Understanding Spoken Commands

We conducted an experiment to evaluate the effect of Seq2Seq-NI on the understanding of robot-directed spoken commands. We also validated the contribution of the attention mechanism to Seq2Seq-NI.

### 3.1. Conditions

The task was to translate a recognized word sequence into command sequences. We used a GPSR sentence generator to generate the English sentences representing robot-directed commands for a service robot in a domestic environment. This generator was used in the GPSR task of the RobCup@Home league held in RoboCup2015. A GPSR sentence generator generates sentences by assigning labels representing items, locations, or rooms to slots in the given frames of commands. In this experiment, a sequence of commands consisting of elements of commands was also generated at the same time using the same labels. An illustrative example is given as follows:

- Frame of a sentence: Go to LOCATION get ITEM exit from ROOM okay.
- Sequence of commands corresponding to the frame: Move (LOCATION) Find (ITEM, LOCATION) Grasp (ITEM) Move (ROOM).
- Generated sentence: Go to kitchen table get dog doll exit from visitor Room okay.
- Correct command sequence: Move (kitchen_table) Find (dog_doll Kitchen_Table) Grasp (dog_doll) Move (visitor_Room).

We used 52 items, 8 rooms, and 9 locations in this experiment, as shown in Table 1. A list of action elements of the robot is provided in Table 2.

**Table 1:** Items, rooms, and locations in the environment.

Items	Green Tea/ Orange Juice/ Brown Tea/ Japanese Tea/ Red Tea/
	Lemon Tea/ Strawberry Juice/ Cup Star/ Cup Noodle/ Seafood Noodle/
	Korean Soup/ Egg Soup/ Onion Dressing/ Japanese Dressing/ Chip Star/
	Long Potato/ Blue Potato/ Red Potato/ Stick Potato/ Bleach/
	Cloth Cleaner/ Dish Cleaner/ Bath Cleaner/ white cup/ pink cup/
	tumbler/ empty ketchup/ filled ketchup/ ground pepper/ salt/
	sauce/ soysauce/ sugar/ canned juice/ empty plastic bottle/
	filled plastic bottle/ cubic clock/ bear doll/ dog doll/ rabbit doll/
	toy car/ toy penguin/ toy duck/ nursing bottle/ apple/
	cigarette/ hourglass/ camera/ rubik's cube/ bell pepper/
	twin bell alarm clock/ spray bottle
Rooms	Dining Room/ Living Room/ Corridor/ Kitchen Room/ Visitor Room/
	bed room/ kitchen/ lobby
Locations	Dining Table/ Dining Sofa/ Sofa/ Living Sofa/ Side Table/
	Living Table/ Kitchen Table/ Bar/ Reception Table

**Table 2:** Definition of action elements of a robot.

**Action element of a robot**	**Explanation**
Move (room or location)	Move to the designated place
Grasp (item)	Grasp an object
Place (item, location)	Place an object at the designated place
Find (item or person, room or location)	Find a target and goal, and move there
Follow (person)	Follow a person
Say (person)	Call a person
Listen (item)	Ask about an object

In total, the number of sentences generated for the training and tests were 10,000 and 100, respectively. The number of lexicons was 110 and the number of phonemes was 84. We used the CMU Pronouncing Dictionary^7^ to represent the English phonemes.

Four male participants (L, W, K, and T) were requested to pronounce the generated sentences once each in a natural domestic environment full of daily noises^8^. The participants L and W are not native English speakers, but are fluent, whereas K and T are neither native English speakers nor fluent in the language. The recorded data were encoded at 16 bits at a sampling rate of 16 kHz. The recorded speech signals were recognized using off-the-shelf ASR systems. For comparison, we used two different ASR systems, namely, the Google Speech API^9^ and CMU sphinx^10^. The WER for each ASR system is shown in Table 3 for reference. It can be seen that the speech of the fluent speakers was recognized more accurately by both ASR systems. The Google Speech API outperformed the CMU Sphinx in most cases. However, the overall performance of the speech recognition was still low. This suggests that the noise in the environment was considerably large for an ASR system.

**Table 3:** SER, WER, and PER of the employed ASR systems.

	**Google Speech API**			**CMU Sphinx**		
**#**	**SER**	**WER**	**PER**	**SER**	**WER**	**PER**
L	1.00	0.40	0.71	1.00	0.77	0.82
W	0.94	0.26	0.16	1.00	0.68	0.42
K	1.00	0.58	0.45	1.00	0.99	0.70
T	1.00	0.48	0.38	1.00	1.04	0.75
Avg.	0.99	0.43	0.43	1.00	0.87	0.67

Examples of the generated sentences, correct command sequences, and speech recognition results are shown in Table 4. Most of the recognition results contain some recognition errors.

**Table 4:** Example sentences generated by the GPSR sentence generator and speech recognition results.

**#**	**Original sentence**	**Google API**	**CMU Sphinx**	**Correct command sequence**
W	Well, go to Sofa take empty ketchup finally come back	ell go to sofa take empty ketchup finally come back	Well go to so that him to do just fine in combat	Move (Sofa) Grasp (empty_ketchup) Move (HERE)
W	Go to Dining Sofa next detect camera take it	Go to dining sofa next detect camera tickets	Go to dining set so far next attacked camera take it	Move (Dining Sofa) Find (camera Dining Sofa) Grasp (camera)
W	Move to living table Grasp bleach leave the apartment please	Move to living table grass Plant leave the apartment please	Move to the b. table grasp financial leave the apartment peace	Move (living table) Grasp (bleach) Move (apartment)
T	Go to sofa take Cup Star put it on living table okay	Go to Suffern take out put it on living table okay	That is so tied to a depth than the teeth being in the o.k.	Move (sofa) Grasp (cup star) Move (living table) Place (cup star)
T	Go to kitchen table move to living table take red tea	Go to kitchen table folding table decorative	Go to teach in tampa the two he became the dignity	Move (Kitchen_Table) Move (Living_Table) Find (Red Tea) Grasp (Red Tea) Move (HERE)

During this experiment, we compared six different methods and a rule-based system. Each of the Seq2Seq-based methods is characterized by whether it has a noise injection, whether it uses an attention mechanism, and based on the type of input, i.e., word or phoneme. The six methods are as follows:

1. Seq2Seq using a word input,
2. Seq2Seq with an attention mechanism and using a word input,
3. Seq2Seq using a phoneme input,
4. Seq2Seq with an attention mechanism and using a phoneme input,
5. Seq2Seq-NI using a phoneme input, and
6. Seq2Seq-NI with an attention mechanism using a phoneme input.

In this study, we assume that the probability of producing an identical symbol is far higher than that of the others in SDM for noise injection.

(9) $$q_{s}(y_{j}|x_{i})=\{\begin{array}{ll} \frac{L(1-\beta )}{\beta L}P_{s} & y_{j}=x_{i}\\ \frac{1}{\beta L}P_{s} & y_{j}\neq x_{i} \end{array}.$$

The parameters of the SDM are *P*_*I*_ = 0.1, *P*_*S*_ = 0.8, *P*_*D*_ = 0.1, and β = 8.0.

For the original Seq2Seq and the Seq2Seq using an attention mechanism, the number of hidden units is 128 for both the encoders and the decoders. The number of layers is 2 for both the encoders and decoders The network weights are uniformly initialized in [−0.1, 0.1]. The networks are trained for 12,000 training steps using plain SGD. The learning rate is 1.0, the mini-batch size is 128, and the dropout ratio is 0.2. The normalized gradient is rescaled whenever its norm exceeds 5.0.

The rule-based system generates a command sequence by finding keywords, e.g., sofa, move, and grasp, from the input word sequence obtained by the ASR system. Therefore, if the ASR system misrecognizes keywords in a sentence, the rule-based system has no chance to generate a correct command sequence.

All data and codes have been uploaded as open datasets and open sources^11^.

### 3.2. Results

We attempted to determine whether the robot could understand the commands given by the users. During this experiment, we considered the understanding to be a success if the robot could translate an input phoneme or word sequence into a ground-truth command sequence. In the following tables, scores of the highest performance are in bold and underlined, and those of the second highest performance are underlined.

The success rate is presented in Table 5. Because of significant speech recognition errors, most of the methods could not infer the correct command sequence. However, Seq2Seq-NI without attention could infer 21% of the utterances correctly, even though the SER of the recognized speech was mostly 1.00, i.e., almost no sentences were recognized perfectly. This shows that Seq2Seq-NI can improve the performance of language understanding even though the ratio at which the sequence of commands can be estimated perfectly remains low. By contrast, there was a decline in the performance of Seq2Seq without a noise injection when the speech recognition results contained errors. A two-sample test for equality of proportions without continuity correction was performed to evaluate the statistical significance of the differences in the success rate of language understanding shown in Table 5. To evaluate the contribution of noise injection, the difference between the overall success rate of 3 and 5, and that of 4 and 6 were tested. Statistical significance at 1% level was found in all cases (*p* = 4.7 × 10^−3^ and 4.0 × 10^−5^ for Google Speech API, and *p* = 2.7 × 10^−5^ and 9.5 × 10^−3^ for CMU Sphinx, respectively). In addition, the results suggest that the attention mechanism also improves the performance. However, the contribution is relatively small compared to that of the noise injection.

**Table 5:** Success rate of language understanding.

				**Google Speech API**					**CMU Sphinx**				
**No**.	**NI**	**Attention**	**Input**	**L**	**W**	**K**	**T**	**Avg**.	**L**	**W**	**K**	**T**	**Avg**.
-	Rule-based system		Word	0.00	0.00	0.00	0.00	0.00	0.00	0.00	0.00	0.00	0.00
1	–	–	Word	0.00	0.00	0.00	0.00	0.00	0.00	0.00	0.00	0.00	0.00
2	–	✓	Word	0.00	0.00	0.00	0.00	0.00	0.00	0.00	0.00	0.00	0.00
3	–	–	Phoneme	0.04	0.40	0.03	0.06	0.13	0.00	0.01	0.00	0.00	0.00
4	–	✓	Phoneme	0.05	0.33	0.08	0.05	0.13	0.01	0.07	0.02	0.00	0.03
5	✓	–	Phoneme	**0.07**	0.51	0.12	0.13	0.21	0.02	0.13	0.02	**0.03**	0.05
6	✓	✓	Phoneme	0.06	**0.60**	**0.16**	**0.14**	**0.24**	**0.03**	**0.18**	**0.03**	0.01	**0.06**

*The bold and underlined values represents the maximal and the second maximal values, respectively*.

For a more detailed comparison, the WERs of the inferred command sequences were calculated and the results are presented in Table 6. The Welch two-sample *t*-test was performed to evaluate the statistical significance of the differences in the WER shown in Table 6. To evaluate the contribution of noise injection, the difference between the overall WERs of 3 and 5, and that of 4 and 6 were tested. Statistical significance at 1% level was found in all cases (*p* = 1.8 × 10^−5^ and 5.7 × 10^−3^ for Google Speech API, and *p* = 5.5 × 10^−9^ and 4.1 × 10^−5^ for CMU Sphinx, respectively).

**Table 6:** WER of inferred command sequences.

				**Google Speech API**					**CMU Sphinx**				
**No**.	**NI**	**Attention**	**Input**	**L**	**W**	**K**	**T**	**Avg**.	**L**	**W**	**K**	**T**	**Avg**.
-	Rule-based system		Word	0.60	0.40	0.65	0.59	0.56	0.72	0.71	0.90	0.86	0.80
1	–	–	Word	0.66	0.69	0.65	0.60	0.65	0.75	0.62	0.62	0.66	0.66
2	–	✓	Word	**0.46**	0.46	0.50	0.49	0.48	0.50	0.50	0.50	0.51	0.50
3	–	–	Phoneme	0.48	0.18	0.44	0.37	0.37	0.59	0.43	0.57	0.58	0.54
4	–	✓	Phoneme	0.48	0.18	0.37	0.35	0.35	0.51	0.38	0.53	0.50	0.48
5	✓	–	Phoneme	0.47	0.08	**0.31**	**0.27**	**0.28**	0.52	0.23	**0.46**	0.46	0.42
6	✓	✓	Phoneme	0.47	**0.07**	0.34	**0.27**	0.29	**0.48**	**0.22**	0.48	**0.42**	**0.38**

*The bold and underlined values represents the minimal and the second minimal values, respectively*.

To qualitatively illustrate the results in greater detail, typical examples are shown in Table 7. The underlined parts indicate the recognition or estimation errors. These examples show that Seq2Seq2-NI, i.e., 5 and 6, improve the language understanding performance even when the speech recognition result is not fully correct. For example, even though “take it” was recognized as “tickets” by the Google Speech API, Seq2Seq2-NI successfully inferred “Grasp (camera)” from the sentence. Although the CMU sphinx recognized “Dining_Sofa” as “dining set so far,” Seq2Seq2-NI inferred “Dining_Sofa” as an element of the command sequence.

**Table 7:** Typical examples.

**Speaker**	**W**	
Original sentence	Go to Dining_Sofa next detect camera take it.	
Label	Move (Dining_Sofa) Find (camera Dining_Sofa) Grasp (camera)	
ASR system	Google Speech API	CMU Sphinx
Speech recognition result	Go to dining sofa next detect camera tickets	Go to dining set so far next attacked camera take it
1	Move (Dining_Room) Find (Cloth_Cleaner Dining_Room) Grasp (Cloth_Cleaner) Move (Dustbin)	Move (Dining_Room) Find (person Dining_Room) Grasp (Japanese_Dressing) Move (Bar) Place (Japanese_Dressing Bar)
2	Move (kitchen) Find (camera kitchen) Move (HERE)	Move (lobby) Find (person lobby) Follow (person)
3	Move (Dining_Sofa) Move (Dining_Table) Find (camera Dining_Sofa) Grasp (camera) Move (HERE)	Move (Sofa) Find (toy_car Sofa) Grasp (toy_car) Move (Bar)
4	Move (Dining_Sofa) Move (Dining_Table) Find (camera Dining_Sofa) Grasp (camera) Move (HERE)	Move (Dining_Sofa) Find (camera Dining_Sofa) Grasp (camera)
5	Move (Dining_Sofa) Find (camera Dining_Sofa) Grasp (camera)	Move (Dining_Sofa) Find (camera Dining_Sofa) Grasp (camera)
6	Move (Dining_Sofa) Find (camera Dining_Sofa) Grasp (camera)	Move (Dining_Sofa) Find (camera Dining_Sofa) Grasp (camera)

*The underlined parts represent errors*.

## 4. Experiment 2: Effect of Noise Injection

We investigated the influence of the hyperparameters of the SDM for noise injection on the overall performance of Seq2Seq-NI in terms of language understanding. Theoretically, a noise injection simulates the recognition errors observed in the results of real speech recognition. If the noise level is 0, Seq2Seq-NI becomes the same as Seq2Seq. However, if the noise level reaches too high, Seq2Seq-NI will not be able to determine the proper relationship between the input and output. Therefore, investigating the impact of hyperparameters such as those of the noise level is crucial. In this study, we conducted an experiment using different settings of the hyperparameters and investigated the relationship among the overall performance of Seq2Seq-NI, the recognition error rate of the ASR systems, and the level of noise injection.

### 4.1. Conditions

The hyperparameters of the SDM have three degrees of freedom corresponding to the insertion, deletion, and substitution. In this study, we focus only on the noise level and ignore the contributions of the characteristics of the three types of deformation. We define ρ as a control parameter of the noise level.

$$\begin{array}{l} P_{I}=P_{D}=\frac{L-1}{\beta L}P_{S}=\rho . \end{array}$$

In this experiment, we use the same datasets and parameters as in Experiment 1, except for the noise level.

### 4.2. Results

**Figure 5:**
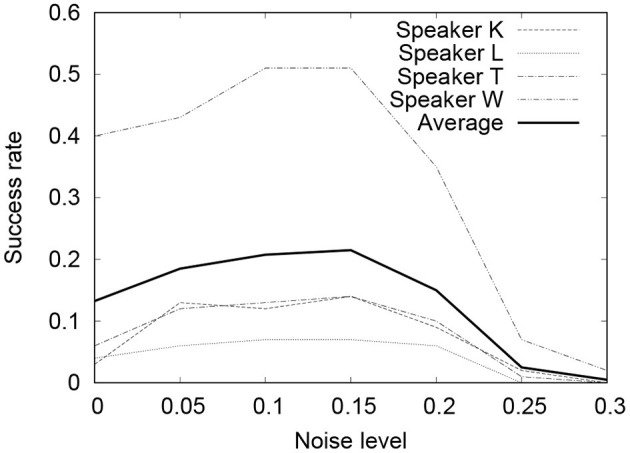
SER of command sequences with different noise levels (Google Speech API).

**Figure 6:**
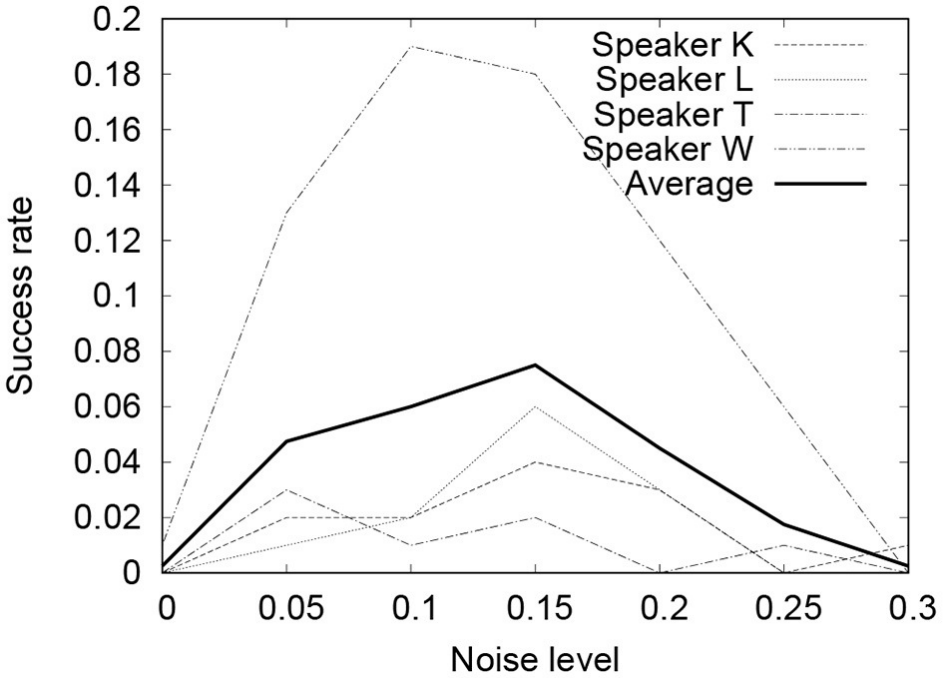
SER of command sequences with different noise levels (CMU Sphinx).

Figures 5, 6 show the results of the experiment. In both cases, when ρ = 0.1 ~ 0.15, the performance of Seq2Seq-NI is better and there are no clear differences between the two. Theoretically, the noise-injected input sentence with 0 has $\text{P}\text{E}\text{R}=\frac{3\rho }{1-\rho }$. Therefore, ρ = 0.1 ~ 0.15 corresponds to **PER** = 0.33 ~ 0.53. This is similar to the PER given in Table 3. This indicates that the performance of Seq2Seq-NI is better when the hyperparameters simulate the actual noise level of the speech recognizer.

## 5. Conclusion

A new method of language understanding for robot-directed spoken commands called Seq2Seq-NI was proposed. The method employs off-the-shelf automatic speech recognition (ASR) systems in a domestic environment. Seq2Seq-NI employs Seq2Seq as a semantic parser of recognized spoken commands. Owing to the nature of a neural network, noise can be injected into the semantic parsing. We employed the SDM to deform or inject noise into a variable-length symbol sequence such as a recognized spoken command. The results of the experiment indicate that Seq2Seq-NI outperforms the baseline methods. Noise injection clearly improves the understanding of spoken commands. It was also shown that an attention mechanism contributes to an improved performance of the semantic parsing. We also conducted an experiment to evaluate the influence of the injected noise. It was found that a noise level simulating the actual recognition error rate of the ASR improves the performance of Seq2Seq-NI.

Despite demonstrating the validity of Seq2Seq-NI, further investigations should be conducted. During the experiment, we assume that the word choices by users are constrained. In a real service robot environment, however, users are unconstrained in their use of words. If a new word is spoken in a sentence, the word is regarded as a type of noise and the sentence is mapped onto a command sequence as a result of a generalization by the neural network. We expect that the noise injection method will improve the performance under scenarios with unconstrained word choices. However, investigating the applicability of the method in cases in which users are allowed to generate unconstrained commands remains as a future challenge.

The understanding of sentences for GPSR, as used in the experiment, is a popular and accepted task in the field of service robotics. However, evaluating our method on other datasets, such as the TrainRobots Dataset (Dukes, 2014), and understanding the characteristics of the method more clearly are essential tasks.

In this study, we focused on a robot command interpretation task. However, the architecture is more general, and is expected to be used for other tasks in which speech recognition results are applied by a neural network-based postprocessing system. Furthermore, in this study, we used a basic off-the-shelf neural network-based semantic parser, i.e., Seq2Seq. The main aim of this study was to demonstrate the validity of noise injection for a neural network-based semantic parser to improve its robustness to recognition errors caused by ASR systems. This implies that a wide range of neural network-based semantic parsers can be adopted for this idea. For example, Eppe et al. (2018) used a dilated causal convolutional neural network for robot command interpretation. Moreover, applying the concept of phoneme-level noise injection to other neural network-based semantic parsers is also a possible task for a future study.

The remaining challenges are as follows: The first is to implement the method in a real service robotics environment and evaluate its performance and validity. The next is to develop an extension of this method to on-line learning. New items and names of persons will be introduced, not only in RoboCup@Home but also in our daily environment. The current Seq2Seq-NI requires additional training of the encoder-decoder network, which may involve significant computational costs. Further, conditional information in language understanding must be considered. If the robot can recognize its current place and an object in front of it, it may be able to use such information to improve its language understanding. This may be achieved by introducing a conditional term into Seq2Seq-NI. In addition, determination of the hyperparameters of the SDM will be important. The results of Experiment 2 indicate that the recognition error level of the ASR system is a key to optimizing the SDM in Seq2Seq-NI. Thus, we must conduct theoretical and experimental investigations.

## Data Availability Statement

The datasets used for this study can be found at our GitHub repository https://github.com/EmergentSystemLabStudent/noise_injection_seq2seq.

## Author Contributions

YT designed the study, collected data and conducted an experiment. HT conducted another experiment. YH contributed to analysis and interpretation of data, and assisted in the preparation of the manuscript. TT wrote the manuscript and contributed to the key idea of this study. All authors approved the final version of the manuscript, and agree to be accountable for all aspects of the work in ensuring that questions related to the accuracy or integrity of any part of the work are appropriately investigated and resolved.

### Conflict of Interest

The authors declare that the research was conducted in the absence of any commercial or financial relationships that could be construed as a potential conflict of interest. The handling editor is currently organizing a Research Topic with one of the authors TT, and confirms the absence of any other collaboration.

## Footnotes

^1^https://cloud.google.com/speech-to-text/

^2^https://cmusphinx.github.io/

^3^https://github.com/julius-speech/julius

^4^SentenceGenerator:github.com/komeisugiura/GPSRsentence_generator

^5^TensorFlow Neural Machine Translation Tutorial https://github.com/tensorflow/nmt

^6^Word-level noise injection eliminates the information regarding the phonological similarity between the original sentence and the output words. A character-level representation is also inappropriate because English letters are not phonetic symbols. Even if the phonetic sequence is entirely the same as in the original, the character sequence is not the same. Based on this idea, in this study, we focused on noise injection at the phoneme level. If the target language has phonetic symbols, such as hiragana letters in Japanese, we can use character-level noise injection.

^7^http://www.speech.cs.cmu.edu/cgi-bin/cmudict

^8^This study was carried out following the Ritsumeikan University Research Ethics Guidelines, Research Ethics Committee of Ritsumeikan University. An ethical review process was not required for this study because none of the conditions for such a review based on the checklist provided by the committee were satisfied. All participants gave written informed consent.

^9^https://cloud.google.com/speech-to-text/?hl=ja

^10^https://cmusphinx.github.io/

^11^https://github.com/EmergentSystemLabStudent/noise_injection_seq2seq
